# Phenotype study of multifoliolate leaf formation in *Trifolium alexandrinum* L.

**DOI:** 10.7717/peerj.10874

**Published:** 2021-03-01

**Authors:** Devendra Ram Malaviya, Ajoy Kumar Roy, Pankaj Kaushal, Shalini Pathak, Ruslan Kalendar

**Affiliations:** 1 ICAR - Indian Institute of Sugarcane Research, Lucknow, India; 2 ICAR - Indian Grassland and Fodder Research Institute, Jhansi, India; 3 ICAR - National Institute of Biotic Stress Management, Raipur, India; 4 Department of Agricultural Sciences, University of Helsinki, Helsinki, Uusimaa, Finland; 5 National Laboratory Astana, Nazarbayev University, Nur-Sultan, Aqmola, Kazakhstan

**Keywords:** Egyptian clover, Genetics, Pentafoliate, Multifoliate and trifoliate plants, The multifoliate trait

## Abstract

**Background:**

The genus *Trifolium* is characterized by typical trifoliolate leaves. Alterations in leaf formats from trifoliolate to multifoliolate, i.e., individual plants bearing trifoliolate, quadrifoliolate, pentafoliolate or more leaflets, were previously reported among many species of the genus. The study is an attempt to develop pure pentafoliolate plants of *T. alexandrinum* and to understand its genetic control.

**Methods:**

The experimental material consisted of two populations of *T. alexandrinum* with multifoliolate leaf expression, i.e.,interspecific hybrid progenies of *T. alexandrinum* with *T. apertum*, and *T. alexandrinum* genotype Penta-1. Penetrance of the multifoliolate trait was observed among multifoliolate and trifoliolate plant progenies. In vitro culture and regeneration of plantlets from the axillary buds from different plant sources was also attempted.

**Results:**

The inheritance among a large number of plant progenies together with in vitro micro-propagation results did not establish a definite pattern. The multifoliolate leaf formation was of chimeric nature, i.e., more than one leaf format appearing on individual branches. Reversal to normal trifoliolate from multifoliolate was also quite common. Penetrance and expression of multifoliolate leaf formation was higher among the plants raised from multifoliolate plants. Multifoliolate and pure pentafoliolate plants were observed in the progenies of pure trifoliolate plants and vice-versa. There was an apparent increase in the pentafoliolate leaf formation frequency over the years due to targeted selection. A few progenies of the complete pentafoliolate plants in the first year were true breeding in the second year. Frequency of plantlets with multifoliolate leaf formation was also higher in in vitro axillary bud multiplication when the explant bud was excised from the multifoliolate leaf node.

**Conclusion:**

Number of leaflets being a discrete variable, occurrence of multifoliolate leaves on individual branches, reversal of leaf formats on branches and developing true breeding pentafoliolates were the factors leading to a hypothesis beyond normal Mendelian inheritance. Transposable elements (TEs) involved in leaf development in combination with epigenetics were probably responsible for alterations in the expression of leaflet number. Putative TE’s movement owing to chromosomal rearrangements possibly resulted in homozygous pentafoliolate trait with evolutionary significance. The hypothesis provides a new insight into understanding the genetic control of this trait in *T. alexandrinum* and may also be useful in other *Trifolium* species where such observations are reported.

## Introduction

The genus *Trifolium*, comprising 290 temperate and subtropical species, is believed to have originated in Asia Minor. Among various important forage and pasture species of the genus, Egyptian clover (*Trifolium alexandrinum* L.) commonly called as Berseem is cultivated as a winter season annual forage crop in the Mediterranean basin, Iraq, Iran, Pakistan, and India (Vaez Zadeh, 1989; Malaviya et al., 2019a).

Leaves of members of Fabaceae are usually pinnately (occasionally trifoliolate) compound. The leaves in the genus *Trifolium* are non-articulate trifoliolate with lanceolate leaflets attached to long petiole. The multifoliolate trait involves plants that express more than the usual three leaflets formats. Alterations in leaf structure from trifoliolate to multifoliolate were reported previously with occasional presence in *T. pratense* (Thaler & Weber, 1955; Laczynska-Hulewicz, 1956) and *T. repens* (Stark, 1926). Unifoliolate *T. repens* was also described by Atwood (1938). The four-leaf clover is recognized worldwide as a symbol of good fortune. A four-leaf white clover, exhibiting approximately 60% heritable expression, was developed through mutagenesis (Song et al., 2009). Numerous ornamental multifoliolate white clover genotypes were also reported by the Margot Forde Germplasm Centre, New Zealand (Richardson et al., 2008). Multifoliolate leaf formation was also reported in diploid (Bingham, 1968), tetraploid (Bingham, 1966; Ferguson & Murphy, 1973), and hexaploid alfalfa (Bingham & Binek, 1969). Multifoliolate leaves in alfalfa were considered to substantially increase the photosynthetic area of the plants (Chatterton, 1976). Multifoliolate leaf formation is also described in some other leguminous crops like groundnut (Vasanthi, 2003), pigeon pea (Pandey & Pawar, 2000), and *mungbean* (Soehendi et al., 2007). Genetic control of multifoliolate leaf formation among different species have also been presented including the recent extensive work by Chen et al. (2010) and He et al. (2020) in *Medicago truncatula*.

*T. alexandrinum* is domesticated in its diploid (2*x* = 16) form (Kumari & Bir, 1990; Malaviya et al., 2004a; Malaviya et al., 2004b; Vizintin, Javornik & Bohanec, 2006), although tetraploid cultivar have also been developed through colchicine induced polyploidy (Roy, Malaviya & Kaushal, 1998; Kaushal et al., 2003). The species possesses trifoliolate leaves; however, occasional occurrence of multifoliolate plants in the natural population was reported (Hasan, 1967; Shukla & Malaviya, 1988). The frequency of such multifoliolate plants in the natural population is only 0.004% and the expressivity of multifoliolate leaves on a plant is 1–2% only. The process of developing multifoliolate Berseem was initiated about two decades ago at ICAR-IGFRI, Jhansi, India by following recurrent phenotypic selection with maintenance of plants in isolation, resulting in plants with high penetrance and or expressivity of the pentafoliolate trait (Indian Grassland and Fodder Research Institute, 2007). Pentafoliolate lines (Penta-1, Black Seeded Pentafoliolate, Tetraploid Pentafoliolate) in *T. alexandrinum* with high expression and penetrance of the trait have been reported (Malaviya, Roy & Kaushal, 2009; Malaviya et al., 2012; Singh et al., 2016; Pathak, 2011; Pathak et al., 2015). The diploid (2*x* = 16) Penta-1 population was developed from a natural multifoliolate variant after repeated selfing and selection for high percent of pentafoliolate leaves for six generations. Black seeded pentafoliolate, a diploid genetic stock, belonged to an interspecific cross between *T. alexandrinum* × *T. apertum*, of which the female parent was multifoliolate (Malaviya et al., 2004a), and lines with high percent of pentafoliolate leaves were developed following recurrent selection for six generation. The third population, i.e., tetraploid pentafoliolate was developed by inducing tetraploidy in Penta-1 population followed with recurrent selection (Pathak et al., 2015). Bakheit (1996) also developed a multifoliolate type of *T. alexandrinum* in Egypt after nine generations of selection from a mutant plant of the single cut ‘Fahl’ cultivar. However, its genetics remained unclear because of complexity of the trait, i.e., occasional appearance of chimeric multifoliolate plant in otherwise trifoliolate population. Additionally, genetic studies were limited owing to non-availability of true breeding plants with other than trifoliolate leaf format. The trait is of importance for increasing biomass yield through increasing photosynthetic area particularly in a crop like *T. alexandrinum,* with a narrow genetic base (Malaviya et al., 2004c; Malaviya et al., 2005; Malaviya et al., 2008; Kumar et al., 2008; Badr, El-Shazly & Watson, 2008; Kaur et al., 2017). Selection for higher leaflet numbers has also been considered as one means for improving protein content and other quality traits in Lucerne (Juan et al., 1993). Hence, this study was conducted to develop pure pentafoliolate plants of *T. alexandrinum* and to understand its genetic control.

## Materials and Methods

The experimental material consisted of the two diploid (2*n* = 16) populations of *T. alexandrinum* with multifoliolate leaf expression, i.e., (i) selfed single plant progenies of Penta-1: INGR 09045 (multifoliolate population of *T. alexandrinum*) (Malaviya, Roy & Kaushal, 2009) and (ii) 58 multifoliolate progenies derived from 11 randomly selected segregating progenies of the interspecific cross *T. alexandrinum* (JHB 146) × *T. apertum* (EC 401712) (Malaviya et al., 2004a), henceforth referred to as ISH-multifoliolate (ISH-multi), in third generation of selfing. Both the parents of this cross combination were diploid. While the female parent JHB 146 is an improved diploid cultivar of *T. alexandrinum,* the male parent EC 401712 is an accession of wild species *T. apertum*. Cultivar JHB 146 is trifoliolate (with rare presence of multifoliolate plants in nature), however for interspecific crossing multifoliolate plant was selected (Malaviya et al., 2004a). EC 401712 was trifoliolate. The seeds were procured from the Gene Bank of the ICAR—Indian Grassland and Fodder Research Institute, Jhansi, India. The healthy seeds were sown in a fly-proof net house at the research farm of the institute for two consecutive years. Sowing was done in 3-m rows at 50-cm intervals. Initially sowing of the harvested seeds from individual plants was done in rows. However, after recording of penetrance data, the plants were randomly uprooted in order to maintain plant-to-plant distance of 15 cm. Thus, there were more plants for penetrance data than the plants for expressivity data. Irrigation and fertilizers were applied as per standard protocol of agronomic practices. The first cutting, 5 cm above ground, was done at 75 days after sowing, and subsequent cuttings at 45-day intervals, with the last cutting on March 15 when the crop was left for flowering and seeds were collected.

Penetrance of the multifoliolate trait was observed at leaf-stage 4–5, and observations were recorded by counting the total number of multifoliolate and trifoliolate plants within individual progenies. Leaflet formation such as trifoliolate, quadrifoliolate, pentafoliolate, hexafoliolate, and heptafoliolate were recorded, on individual plants in each progeny, at each nodal position among all the plants two times, i.e., before the first cutting, and 45 days after the second cutting on regenerated branches. Hand tripping of flowers was done to enhance the seed set in flowers, which is normally very low in the absence of a bee pollinator (Roy, Malaviya & Kaushal, 2005).

### In vitro axillary bud multiplication studies

The study was carried out in two populations in a popular diploid cultivar of *T. alexandrinum* Wardan, and diploid Penta-1. Plants were raised through seeds for 50–60 days up to 4 to 5 nodes stage under aseptic conditions on MS media (Murashige & Skoog, 1962) with 0. 3% sucrose and solidified by adding 0.7% agar. The healthy plants grown under aseptic conditions were carefully removed from the media, and the axillary buds, 3-to5-mm-long stem pieces, from the different nodes bearing trifoliolate, quadrifoliolate and pentafoliolate leaves were excised for in vitro culture. These explants were cultured on LSP3 media for shoot bud multiplication and RL media for root induction following Roy et al. (2004), and Phillips & Collins (1984). The cultures were maintained at 25  ± 2 °C with a day light cycle of 8–10 h, increased to 10–12 h after shoot emergence. Observations were recorded on 28th day for number of leaves with a variable number of leaflets. The shoots were split and sub-cultured in 4–5 tubes after 30 days as per the availability of plantlets either into root-inducing media (RL) or shoot-induction media (LSP3) or MS basal media. The sub-culturing was done for a total of nine cycles, and observations for expression of the multifoliolate trait were recorded in each culture cycle.

### Results & Discussion

### Penetrance and expressivity of multifoliolate trait

**Figure 1:**
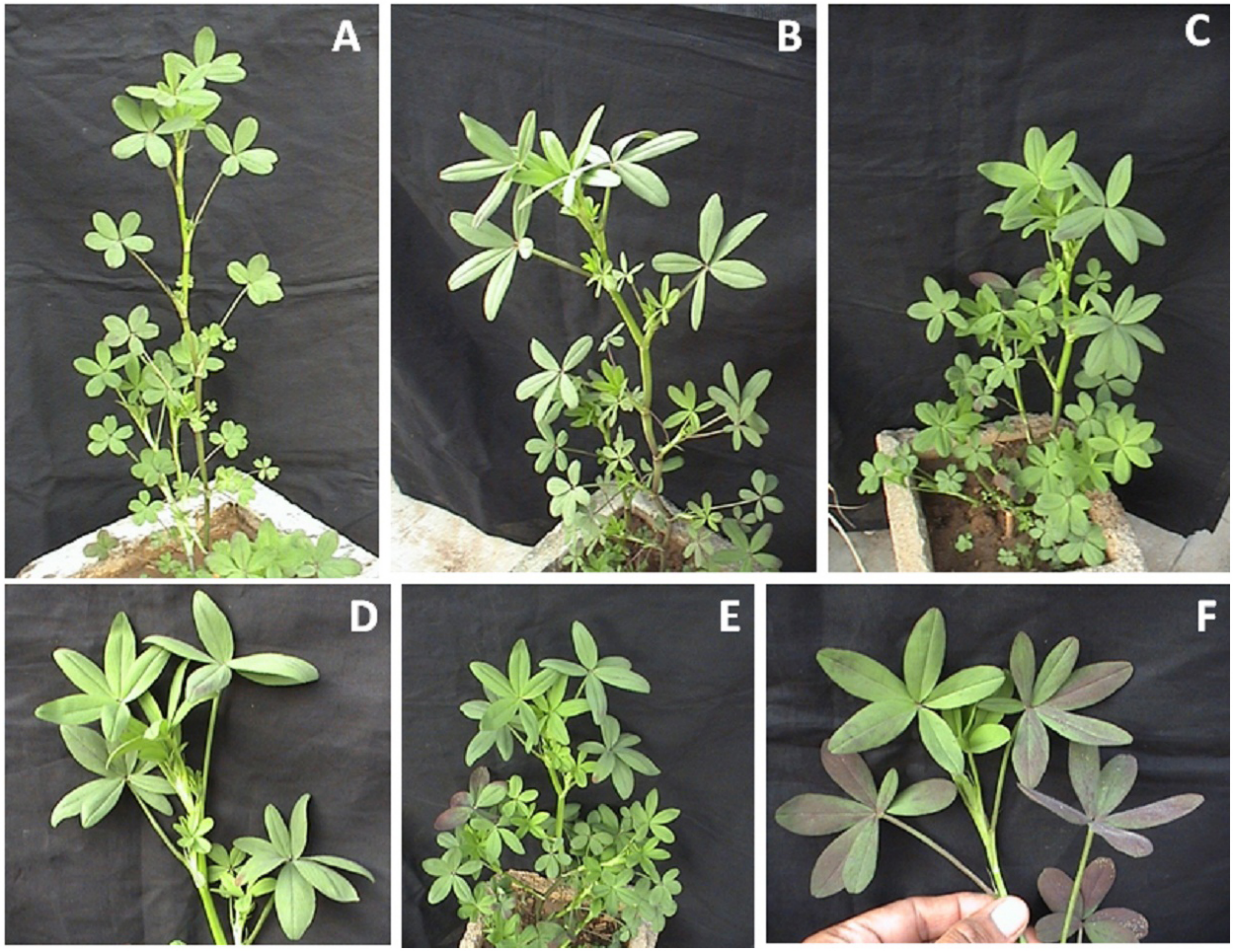
(A–F) Multifoliolate and pentafoliolate plants of Trifolium alexandrinum. (A) Pentafoliolate plant of Penta-1 population. (B) Pentafoliolate plant of ISH-multi population. (C) Multifoliolate plant of ISH-multi population with mostly pentafoliolate leaves and a heptafoliolate leaf. (D) A branch of multifoliolate plant of ISH-multi population with pentafoliolate and heptafoliolate leaves. (E) Multifoliolate plant of ISH-multi population with mostly pentafoliolate leaves and a hexafoliolate leaf. (F) A branch of multifoliolate plant of ISH-multi population with pentafoliolate and hexafoliolate leaf.

The penetrance of multifoliolate trait was observed in two populations, i.e., Penta-1 and ISH-multi. In the first year, 30 self-fertilized progenies of Penta-1 plants exhibited 93.1% to 100% penetrance of multifoliolate leaves. Of these progenies, 18 showed 100% penetrance of the multifoliolate trait (Table 1, Fig. 1), whereas none of the progeny was completely trifoliolate. Among 45 progenies derived from multifoliolate ISH-multi plants, 22 showed 100% expression of multifoliolate leaf formation with no progeny being completely trifoliolate. However, all 13 progenies derived from trifoliolate ISH-multi, were completely trifoliolate (Table 1, Fig. 1). Average penetrance of multifoliolate trait was 80.4% among the progenies of self-fertilized progenies of multifoliolate plants. The plants, in both the populations, marked for high expression of pentafoliolate trait, multifoliolate and trifoliolate leaves were selfed and advanced for next generation.

**Table 1:** Mean and range of penetrance and expression of multifoliolate trait in *Trifolium alexandrinum*.

Plant type	Progenies	Plants	Penetrance^*^ (Progeny average and range)	Progenies with 100% penetrance	Progenies with Nil penetrance	Expressivity of leaf formats											
						Expression before first cut						Expression after second cut					
						Plants	T	Q	P	Hx	Hp	Plants	T	Q	P	Hx	Hp
Penta 1—First year																	
M	30	1487	98.6 (93.1-100)	18	0	499	16.7 (11.9–46.1)	9.4 (4.5–19.6)	73.4 (41.9–92.1)	0.3 (0-2.7)	0.1 (0-1.8)	418	6.3 (0-21.9)	4.6 (0-9.6)	88.5 (73.2–99.5)	0.6 (0-7.7)	0.1 (0-1.0)
Penta 1—Second year																	
P	49	827	85.6 (16.7-100)	16	0	512	17.0 (0–100)	8.97 (0-57.1)	73.3 (0–100)	0.6 (0-33.3)	0.1 (0.3-0)	512	4.8 (0–100)	1.4 (0-44.8)	93.6 (0–100)	0.1 (0-25.6)	0.02 (0-2.3)
M	37	826	88.8 (57.1-100)	12	0	362	13.3 (0–100)	8.2 (0-60.0)	77.9 (0–100)	0.3 (0-30.0)	0.3 (0-26.7)	362	1.5 (0–100)	1.6 (0-40.7)	96.6 (1–100)	0.2 (0-28.5)	0.04 (0-8.1)
T	2	72	63.4 (48.3–78.6)	0	0	24	18.9 (0–100)	12.8 (0-55.6)	68.3 (0–100)	0	0	24	6.6 (0–100)	1.3 (0-11.1)	92.1 (0-100	0	0
ISH –multi-First year																	
M	45	459	80.4 (14.3-100)	22	0	459	54.9 (0–100)	13.8 (0-66.7)	31.3 (0-83.3)	0.01 (0-20.0)	0	90	18.4 (0–100)	7.7 (0-50.0)	73.7 (0–100)	0.3 (0-28.6)	
T	13	38	0	0	13	38	100	0	0	0	0	14	63.5 (0–100)	3.2 (0-22.2)	33.3 (0–100)	–	
ISH-multi-Second year																	
P	11	73	97.6 (85–100)	9	0	73	20.9 (0–100)	9.3 (0-60.0)	69.8 (0–100)	–		73	2.4 (0–100)	3.5 (0-35.7)	93.6 (0–100)		
M	26	96	80.3 (0–100)	17	3	96	42.8 (0–100)	8.74 (0-55.6)	48.7 (0–100)	0.4 (0-14.3		96	26.8 (0–100)	5.1 (0-34.0)	68.1 (0–100)		
T	13	48	79.8 (33.3-100)	6	0	48	49.2 (0–100)	12.3 (0-50.0)	38.5 (0–100)	–		47	26.4 (0–100)	8.8 (0-42.3)	64.9 (0–100)		

**Notes.**

M multifoliolate T Trifoliolate P Pentafoliolate Q Qudrifoliolate Hx Hexafoliolate Hp Heptafoliolate

* Figures in parenthesis are range values

In second year of observation, among selfed plants derived from near pentafoliolate (plants with >95% pentafoliolate leaves), multifoliolate and trifoliolate plants of Penta-1 population, average penetrance of the multifoliolate trait across the progenies was 85.6%, 88.8% and 63.4 respectively (Table 1). Among 49 progenies derived from pentafoliolate plants of Penta-1 in the first year, 16 progenies showed 100% penetrance of multifoliolate leaf formation whereas remaining progenies were noted for presence of some multifoliolate plants (Table 1). Similarly, 12 out of 37 self-fertilized progenies, derived from multifoliolate plants, showed 100% penetrance of multifoliolate leaf formation. The two trifoliolate progenies were also noted for multifoliolate leaf formation with 48.3 and 78.6% penetrance. Among 11 progenies derived from pentafoliolate plants of ISH-multi in the first year, 9 progenies showed 100% penetrance of multifoliolate leaf formation whereas remaining progenies were noted for presence of some multifoliolate plants (Table 1). Similarly, 17 out of 26 progenies, derived from multifoliolate plants, showed 100% penetrance of multifoliolate leaf formation. Three progenies of this population were completely trifoliolate also. Interestingly, none of the progenies, derived from multifoliolate ISH-multi plants in the first year, was completely trifoliolate, rather six progenies were noted for 100% penetrance of multifoliolate leaf formation (Table 1). In the ISH-multi population, the selfed plant progenies of near pentafoliolate, multifoliolate, and trifoliolate plants revealed 97.6%, 80.3% and 79.8% average penetrance respectively.

Expressivity of the traits recorded as percentage of multifoliolate leaves on individual plants revealed 73.4% expressivity of pentafoliolate along with a few quadrifoliolate, hexafoliolate and heptafoliolate leaves among progenies of Penta-1 population before the imposition of the first cutting (Table 1, Fig. 1) and only 16.7% leaves were trifoliolate. Of the 499 plants studied, 18 were pure trifoliolate and 106 were near pentafoliolate (>90% pentafoliolate leaves). Among these progenies, one progeny was noted with the highest frequency (58%) of pure pentafoliolate plants whereas a few other progenies did not show any pure pentafoliolate plants. In all, seven progenies were identified with high expression of pentafoliolate leaves. After the second cutting of Penta-1 plants progenies, the appearance of pentafoliolate leaves increased further along with a decrease in trifoliolate leaf appearance (Table 1). Other leaf formats were almost negligible. Among 418 plants studied after second cutting, 10 pure trifoliolate and 317 pentafoliolate or near pentafoliolate plants were observed. Progeny 06-13 was observed to have 3 pure trifoliolate and 9 pure pentafoliolate plants whereas, 2 progenies (06-16 and 06-17) were identified with 17 pentafoliolate plants. Maximum expressivity of pentafoliolate leaves in any progeny was 99.5%. Expressivity of multifoliolate trait, pentafoliolate in particular, after second cutting of these plants increased along with a decrease in trifoliolate and other leaf formats (Table 1).

In the second year of observations, the near pentafoliolate, multifoliolate, and trifoliolate selfed plant progenies of Penta-1 genotype did not show much difference for expressivity of multifoliolate trait. The expressivity of pentafoliolate leaves were 73.3%, 77.9% and 68.3% in near pentafoliolate, multifoliolate, and trifoliolate plant progenies respectively before cutting which increased to 93.6%, 96.6% and 92.1% respectively after the second cutting (Table 1). Some multifoliolate plants showed 100% pentafoliolate expression after the second cutting. The expressivity of pentafoliolate leaves after second cutting increased substantially, and many pentafoliolate plants were observed among all three categories of plants. Out of 88 progenies 57 were noted for 100% expressivity of pentafoliolate leaves. After second cutting, 781 complete pentafoliolate plants were identified.

As regards expressivity of multifoliolate trait in plants of ISH-multi population, out of total 497 plants, 153 were pure trifoliolate and 16 near pentafoliolate. Expressivity of multifoliolate trait among plant progenies of multifoliolate plants was 31.3% pentafoliolate with 54.9% trifoliolate leaves which changed to 73.7% and 18.4% respectively after second cutting (Table 1). ISH-multi progenies of trifoliolate plants did not express multifoliolate trait before cutting, however, after second cutting, formation of 33.3% pentafoliolate and 63.5% trifoliolate leaves was noted. Progeny 04-39-25 was observed with 50% near pentafoliolate plants in contrast to progeny 04-47-3 with 65% pure trifoliolate plants. Two trifoliolate progenies became 100% pentafoliolate after the second cutting. Out of 30 progenies, 10 were noted for 100% expressivity of pentafoliolate leaves. Among 104 plants studied after second cutting, 17 pure trifoliolate and 53 pure pentafoliolate plants were observed.

In the second year of observation, the expressivity of multifoliolate trait among selfed progenies of near pentafoliolate, multifoliolate, and trifoliolate plants of ISH-multi, before first cutting, showed wide differences. The expressivity of pentafoliolate leaves ranged from 69.8 to 38.5% among the three groups before first cutting and from 93.6 to 64.9% after second cutting. Expressivity of pentafoliolate leaves was high among plants derived from near pentafoliolate plants and low among plants derived from trifoliolate plants (Table 1). Some trifoliolate plants became multifoliolate after cutting, whereas the majority remained trifoliolate. Out of 50 progenies 10 were noted for 100% expressivity of pentafoliolate leaves. After second cutting, 101 pentafoliolate plants were identified.

The expressivity of the multifoliolate trait at different nodal positions was observed in the two populations for two years and the two growth stages, i.e., before first cutting and after second cutting. It was a general trend that before the first cutting, occurrence of trifoliolate leaves was higher at lower nodes which decreased gradually towards the upper nodes whereas expressivity of the multifoliolate trait showed gradual increase (Table 2). The expression of trifoliolate leaves after the second cutting also followed the same trend; however, the expressivity of pentafoliolate leaves increased at all the nodes. The formation of quadrifoliolate leaves also tends to decrease from 1st nodal position to the last whereas other leaf formats like hexafoliolate and heptafoliolate increased (Table 2).

**Table 2:** Leaf formats at different nodal positions (%) in *Trifolium alexandrinum*.

		Leaf formats at different nodal positions (%)											
Progeny detail		N1^*^	**N2**	**N3**	**N4**	**N5**	**N6**	**N7**	**N8**	**N9**	**N10**	**N11**	**N12**
Penta-1—first year													
Before First cut	Tri	50.5	29.5	17.6	10.4	7.1	5.2	3.8	3.7	2.8			
	Quadri	21.2	22.4	10.8	7.8	5.2	4.4	2.7	1.7	0.0			
	Penta	28.3	47.9	71.3	81.8	87.1	89.7	92.4	92.8	96.7			
	Hexa	0.0	0.2	0.2	0.0	0.4	0.4	0.9	1.2	0.6			
	Hepta	0.0	0.0	0.0	0.0	0.2	0.2	0.2	0.6	0.0			
After second cut	Tri	8.7	6.2	5.9	5.4	4.5	4.5	3.3	3.0	1.6			
	Quadri	6.3	6.5	5.0	4.2	3.3	2.6	2.3	0.6	0.0			
	Penta	84.9	87.2	88.9	90.0	91.5	91.6	93.2	95.4	97.3			
	Hexa	0.1	0.1	0.2	0.4	0.7	1.3	1.2	1.0	1.1			
Penta-1—second year													
Before first cut	Tri	69.7	29.2	11.1	8.3	5.0	4.2	3.7	4.2	4.2	3.9	4.0	2.5
	Quadri	12.4	27.1	16.9	8.4	5.4	3.4	2.9	1.8	1.8	2.6	0.9	1.2
	Penta	17.9	43.8	71.7	82.9	88.8	91.5	91.7	92.3	92.2	91.2	93.9	95.1
	Hexa	0.0	0.0	0.1	0.2	0.7	0.6	1.2	1.3	1.2	1.8	1.2	1.2
	Hepta	0.0	0.0	0.1	0.2	0.1	0.3	0.6	0.5	0.6	0.6	0.0	0.0
After second cut	Tri	6.3	4.1	4.1	3.8	3.4	3.5	3.3	3.3	2.8	2.9	2.4	2.0
	Quadri	2.5	3.7	2.9	2.4	2.0	1.4	1.2	1.0	0.7	0.3	0.2	0.2
	Penta	91.2	92.1	93.0	93.8	94.5	95.0	95.3	95.6	96.3	96.5	96.9	97.4
	Hexa	0.0	0.0	0.0	0.1	0.1	0.1	0.1	0.2	0.2	0.2	0.3	0.2
	Hepta	0.0	0.0	0.0	0.0	0.0	0.0	0.0	0.0	0.0	0.0	0.1	0.1
ISH-multi -first year													
Before First cut	Tri	86.3	78.4	56.6	41.4	35.2	31.4	10.0					
	Quadri	7.9	9.5	14.1	14.6	9.6	14.3	20.0					
	Penta	5.8	12.1	29.3	44.0	54.6	54.3	70.0					
	Hexa	0.0	0.0	0.0	0.0	0.6	0.0	0.0					
After second cut	Tri	37.2	25.1	25.7	17.7	15.3	17.6						
	Quadri	4.5	11.2	7.2	5.7	1.7	0.9						
	Penta	58.3	63.7	67.1	76.6	83.0	80.6						
	Hexa	0.0	0.0	0.0	0.0	0.0	0.9						
ISH-multi -second year													
Before First cut	Tri	81.0	62.6	37.7	23.1	19.1	16.4	10.6	12.6	11.2	9.3	0.0	
	Quadri	6.9	15.9	14.0	19.8	8.8	6.7	8.3	4.0	4.0	1.2	6.4	
	Penta	12.0	21.5	47.9	56.6	71.6	76.4	80.0	82.8	83.2	88.4	93.6	
	Hexa	0.0	0.0	0.5	0.5	0.5	0.5	1.1	0.7	1.6	1.2	0.0	
After second cut	Tri	28.7	21.6	19.8	15.7	15.7	15.4	16.5	14.9	15.0	14.8	10.4	9.6
	Quadri	5.6	7.1	7.3	8.4	6.4	7.0	4.7	5.8	4.0	3.1	3.2	0.6
	Penta	65.8	71.3	72.9	75.8	77.9	77.7	78.8	79.3	80.9	82.1	86.4	89.8

**Notes.**

Tri Trifoliolate Quadri Quadrifoliolate Penta Pentafoliolate Hexa Hexafoliolate Hepta Heptafoliolate

* N1, N2,... denotes node numbers starting from base

Perusal of leaf formation on individual branches showed inconsistency with regard to trifoliolate and multifoliolate leaf formats. There were frequent changes from trifoliolate to multifoliolate and their reversions, thus developing chimeric sectors on individual branches of a plant. Formation of a leaf on each node was considered as a single event and any change in leaf format compared to its preceding node was counted as change or reversal in event in this study. Thus, among the plants of Penta-1, up to 8th node formation in the first year, 3,745 events were observed; of which 686 events were of changes or reversal of leaf formats (Table 3). In the same population after second cutting out of 7,502 events, 1,117 were events of change or reversal. The changes were more from trifoliolate to quadrifoliolate and pentafoliolate leaf formats, and from quadrifoliolate to pentafoliolate (Table 3). The reversals were frequently observed from pentafoliolate to trifoliolate and quadrifoliolate. Among plants of ISH-multi, out of 2,322 events observed up to 6th node stage in the first year, 494 events were noted for change or reversal in event; although after second cutting, only 139 change or reversal in event were noted out of total 1,198 events. As a whole, the change or reversal events were more frequently effecting a change of single leaflet number than change of two or more leaflets, indicating that some sort of sequential slicing of genes was taking place (Table 3).

**Table 3:** Trifoliolate to multifoliolate and reversal of event, i.e., number of leaflets per leaf compared to preceding nodal positions, in *Trifolium alexandrinum*.

		Change or reversal of event in leaf formats														
Nodes	Events^a^	3^b^ to 4	3 to 5	3 to 6	4 to 3	4 to 5	4 to 6	5 to 3	5 to 4	5 to 6	5 to 7	6 to 3	6 to 5	6 to 7	7 to 6	Total
Penta-1—Before first cutting																
8	3745	121	177	0	29	260	1	13	72	7	2	0	2	1	1	686
Penta-1—After second cutting																
7	7502	100	245	0	70	285	0	100	313	3	0	0	1	0	0	1117
ISH-multi—Before first cutting																
6	2322	129	135	1	33	128	0	11	5	1	0	1	0	0	0	494
ISH-multi—After second cutting																
6	1198	33	33	0	17	36	0	7	12	1	0	0	0	0	0	139

**Notes.**

a Leaf formation at each node considered as one event.

b Refers to number of leaflets per leaf.

The progenies derived from trifoliolate plants versus multifoliolate plants showed variable penetrance of the trait, with progenies of the multifoliolate plant having a higher degree of penetrance. Expressivity of the multifoliolate trait was also greater among the progenies of multifoliolate plants. Similarly, the progenies with high pentafoliolate leaves showed high penetrance and expressivity of the pentafoliolate trait probably because the oligogenes involved in the expression of the trait with incomplete penetrance had variable expressivity. Multifoliolate plants also showed segregation for the pentafoliolate trait, which increased in the advancing generation with selfing and selection. Out of two populations studied Penta-1 and ISH-multi; the results established no marked difference for expressivity of multifoliolate leaf formation among the two populations which could be attributed to the fact that *T. apertum* is closely related to *T. alexandrinum* and is a probable progenitor (Bobrov, 1947; Malaviya et al., 2004b; Malaviya et al., 2008; Malaviya et al., 2018; Malaviya et al., 2019a; Malaviya et al., 2019b).

### Effect of growth stage and environment on multifoliolate expressivity

Expressivity of the pentafoliolate trait was recorded first during cooler climate, i.e., 75 days after sowing before the first cut (average sunshine 8.48 hours, min temperature 4.8–13.2 °C, max temp 21.4–31.5 °C, morning Relative Humidity (RH) 85%, afternoon RH 38%); and then during warmer climate, i.e., 45 days after the second cutting (average sunshine 10.09 hours, max temperature 29.5–42.3 °C, min temperature 12.90–22.6 °C, morning RH 64%, afternoon RH 24%). Expressivity of the pentafoliolate trait increased after the second cutting, as evidenced in the results. Expressivity of pentafoliolate trait in the first year of the Penta-1 population increased from average 73% to 88.47% after the second cutting. The same was also reflected in the range, which was 41.9–92.1 and 73.2–99.5% before the first cutting and after the second cutting respectively (Table 1). Similarly, in the second generation, expressivity of the trait further increased from 73.3% to 93.6% among the progenies derived from pentafoliolate plants (Table 1). Expressivity of the pentafoliolate trait in the first year of the ISH-multi population increased from average 31.3% to 73.7% after the second cut, whereas in the second year, expressivity of the trait increased from average 69.8% to 93.6% after the second cut among the progenies derived from pentafoliolate plants (Table 1). During the two observations, i.e., before first cutting and after second cutting regimes, the crop grows through mild winter to winter and finally in a warmer climate. The ability of the plant to alter its morphology in response to changes in the environment can also be regarded as phenotypic plasticity, which is an important feature of species adaptation (Bradshaw, 1965; Schlichting, 1986; Watson, Hay & Newton, 1997). Mahmood & Abbas (2003) also observed that in T. alexandrinum leaflet traits were significantly influenced by environmental conditions although these changes were more related to overall compactness of leaflets, indicating plasticity in leaf formation. Leaf appearance rate and time to canopy expansion in four annual clover species (arrowleaf, balansa, gland and Persian) has been reported to have slower rate of leaf production with shortening day lengths at emergence than spring-sown crops (Nori, Moot & Black, 2016). Some of the features of leaf development are regular features of shoot development and are components of a genetically regulated program of shoot maturation, whereas other features are plastic and are controlled by the physiological status of the plant and other environmental factors (Allsopp, 1967). Thus, during higher temperature and longer days, the expressivity of the multifoliolate trait increased. However, these results are in contrast to the work by Juan et al. (1993) who reported that shorter photoperiods increase the proportion of multifoliolate leaves more than long photoperiods. Mahmood & Abbas (2003) reported that leaflet traits show plasticity and change with environmental conditions in T. alexandrinum, however, no change in leaflet number was observed. Mokeyeva (1940) reported that in alfalfa, multifoliolate appearance increases in early spring and fall. Our results are in line with the findings of Volenec & Cherney (1990) that leaves produced during early re-growth have fewer leaflets per leaf than more mature re-growth.

### In vitro axillary bud multiplication

Clonal propagation has been a widely used method to develop true to type plants. In many horticultural crops it is done by vegetative propagation. The species, propagated through seeds can also be vegetatively propagated through tissue culture methods. Through micropropagation, it is possible to regenerate new plants from small pieces of plant tissue. The process can be utilized both for developing same genotype and also to regenerate plants from the mutated cells. In the present study, in vitro culture was used as an alternative approach to examine the leaf phenotype stability. Thus, the study aimed to get true to type regenerants of plantlets with uniform leaf format other than the normal trifoliolate. Lee (2007) has used in vitro culture method to develop four-leaf white clover by in vitro culturing of the nodal portion of the stolon and inducing mutation therein before regeneration of plantlets; and selecting the mutants with four leaflets. In the present study, it was aimed to fix the mutation, if any, at different nodal positions. Hence, axillary buds having different leaf formats from three plant sources such as trifoliolate, multifoliolate and pentafoliolate were taken as explants for in vitro culture. Plantlets developed from these explants were repeatedly sub-cultured through nine passages in different media, to see whether such mitotic changes take place and lead to homogeneous expression during in vitro culture also. Axillary buds taken from the trifoliolate plant of *cv* Wardan cultured in shoot induction medium resulted in 100% of the shoots with trifoliolate leaves in the first culture followed by subcultures in two passages of root induction and three passages of shoot induction media. However, during the 6th sub-culture in root induction medium, 2.4% quadrifoliolate leaves and 1.2% pentafoliolate leaves were observed (Table 4). Thus, trifoliolate explants from cv Wardan produced almost trifoliolate leaves only. When pentafoliolate axillary bud from Penta-1 was cultured, the average expressivity of pentafoliolate trait over the cycles of sub-cultures was 81%. In the 6th sub-culture stage, some shoots with trifoliolate leaves were deliberately selected for further sub-culturing to determine whether the multifoliolate leaflet character is masked in them or not. In subcultures, the shoots showed formation of multifoliolate leaves. In root induction media, the expression of pentafoliolate leaflets was very high (Table 4). The average expressivity of pentafoliolate trait was 48% when pentafoliolate axillary bud was taken from the multifoliolate plant, with the highest frequency (72.1%) noted in the 8th sub-culture stage.

**Table 4:** Expressivity of multifoliolate trait (%) in recurrent cultures of axillary buds of *Trifolium alexandrinum*.

Culture in different media													
Source plant (Explants type)		1 C	2 SC		3SC		4 SC	5 SC	6 SC	7SC	8 SC	9 SC	Average (%)
		LSP3	RL	RL	LSP3	MS	LSP3	RL	RL	LSP3	LSP3	RL	
Wardan-Trifoliolate (T)	Tri	100.0	100.0	100.0	100.0		100.0	100.0	96.4	100.0			99.6
	Quadri	0.0	0.0	0.0	0.0		0.0	0.0	2.4	0.0			0.3
	Penta	0.0	0.0	0.0	0.0		0.0	0.0	1.2	0.0			0.2
Penta 1-Pentafoliolate (P)	Tri	9.0	5.8	4.2	4.7	14.1	11.9	14.2	26.6	22.7	0.0	0.0	10.3
	Quadri	11.8	7.2	3.1	9.7	3.1	10.5	15.7	22.5	6.8	1.9	0.0	8.4
	Penta	79.2	86.3	92.6	85.6	76.3	77.6	70.1	50.9	70.5	98.1	100.0	80.7
	Hexa	0.0	0.7	0.0	0.0	5.0	0.0	0.0	0.0	0.0	0.0	0.0	0.5
	Hepta	0.0	0.0	0.0	0.0	1.6	0.0	0.0	0.0	0.0	0.0	0.0	0.1
Penta 1-Multifoliolate (P)	Tri	33.8	26.4	30.6	30.9	31.7	52.0	42.6	55.3	45.6	17.9		36.7
	Quadri	17.1	11.1	15.1	5.5	12.0	17.1	22.2	20.0	14.0	0.0		13.4
	Penta	47.3	59.6	52.5	63.6	56.3	30.2	34.6	24.4	40.4	72.1		48.1
	Hexa	1.6	2.5	1.2	0.0	0.0	0.7	0.3	0.3	0.0	10.0		1.7
	Hepta	0.2	0.4	0.6	0.0	0.0	0.0	0.2	0.0	0.0	0.0		0.1
Penta 1-Multifoliolate (Q)	Tri	33.0	17.1	22.1			48.6	42.8	40.6	61.5			38.0
	Quadri	13.8	16.4	23.5			5.4	26.1	14.6	23.1			17.6
	Penta	49.1	54.0	42.3			34.9	29.7	44.9	15.4			38.6
	Hexa	3.0	10.6	12.0			11.1	1.4	0.0	0.0			5.4
	Hepta	1.0	1.9	0.0			0.0	0.0	0.0	0.0			0.4
Penta 1-Multifoliolate (T)	Tri	47.1	35.3	52.2	36.4	50.0	61.5	51.4	47.4	54.8	0.0	0.0	39.6
	Quadri	22.9	17.9	5.6	9.1	21.4	11.4	20.9	31.2	36.4	7.7	0.0	16.8
	Penta	30.0	46.9	42.2	54.6	28.6	27.1	27.7	21.5	8.8	92.3	100.0	43.6
Penta 1-Trifoliolate (T)	Tri	56.5	47.7	67.6		61.9	95.5	79.8	96.0	13.0	18.2		59.6
	Quadri	26.1	26.4	19.7		7.1	2.3	11.5	2.8	4.4	10.5		12.3
	Penta	17.4	25.9	12.7		31.0	2.3	8.8	1.3	82.6	71.3		28.1

**Notes.**

C Culture SC Subculture Tri Trifoliolate Quadri Quadrifoliolate Penta Pentafoliolate Hexa Hexafoliolate Hepta Heptafoliolate

2SC, 3SC,... denotes cycle of sub-culture.

Quadrifoliolate axillary bud as explant from multifoliolate plant under different media conditions had the average expressivity of pentafoliolate 39%, 38%, 18%, 5% and 0.4% for trifoliolate, quadrifoliolate, hexafoliolate, and heptafoliolate leaflets, respectively. Thus, expressivity of quadrifoliolate leaves was on average high when the axillary bud taken was quadrifoliolate. The average expressivity of pentafoliolate, trifoliolate, and quadrifoliolate leaf was 44%, 40%, and 17% respectively, when trifoliolate axillary bud from multifoliolate plant was used as explants, however, it was 28%, 60% and 12% respectively, when trifoliolate axillary bud was taken from trifoliolate plant.

The in vitro shoot bud multiplication revealed that the frequency of pentafoliolate leaves was higher on the shoots regenerated from the pentafoliolate axillary bud from pentafoliolate plant of Penta-1 than in those generated from a pentafoliolate axillary bud from a multifoliolate plant of Penta-1, indicating that the expression of the trait is genotype-specific. Lee (2007) has also used tissue culture to develop and patent a variety of four-leaf white clover, although it was non-transmissible. Apart from development of various leaf formats during in vitro culture, the in vitro axillary bud multiplication was successful following protocol of Roy et al. (2004) and Phillips & Collins (1984). Regeneration from shoot meristems has been reported in *T. subterraneum* and *T. resupinatum* (Collins, Taylor & Parrot, 1982; Oelck & Schieder, 1983). Regeneration of plantlet in *T. pratense, T. resupinatum* and *T*. *subterraneum* through embryogenesis was reported by Maheswaran & Williams (1986). *T. pratense* was also regenerated by shoot induction from vegetative meristems (Collins, Taylor & Parrot, 1982) and organogenesis from callus (Phillips & Collins, 1979). Regeneration of plantlets through organogenesis in *T. resupinatum, T. apertum* and *T. glomeratum* is also reported (Roy et al., 2005; Malaviya et al., 2006; Kaushal et al., 2006).

### Simple and compound leaf formation

*Trifolium* leaf is typically trifoliolate, i.e., the leaflets arising from a single point without stalk. Simple and compound leaves may develop by fundamentally different mechanisms. *KN1 or KNAT1* gene, either through misexpression in leaf primordia or constitutive expression is reported to have a role in compound leaf formation (Smith et al., 1992; Sinha, Williams & Hake, 1993; Lincoln et al., 1994; Schneeberger et al., 1995; Chuck, Lincoln & Hake, 1996; Hareven et al., 1996). Genes of the class 1 (*KNOX1*) family are involved in compound leaf development in many plants (Champagne & Sinha, 2004). Further, considering leaf complexity in the legumes, the ancestral state is likely to be compound (Champagne et al., 2007). Thus, it is possible that in plants with higher expression of these genes, the leaves tend to be multifoliolate. Moving from trifoliolate to pentafoliolate appears to be a step towards compounding of the leaf. Occasionally, pentafoliolate leaves with rachis formation were also noticed in the present study. Such pinnate leaves appeared to be a step from simple leaf to compound leaf development. An odd leaflet number was preferred over an even number, possibly to have one leaflet at the end (typical Paplionaceous pinnate compound leaf). This reflects the evolutionary step from simple to compound leaves, which might have occurred through increased *knox1* gene expression as postulated by Miller et al. (2006). Compound leaf development among the majority plants in Fabaceae is linked with KNOX 1 gene expression, however, a large sub-clade with inverted repeat-lacking clad (IRLC) lack KNOX1 expression (Wojciechowski et al., 2000), although *Medicago sativa* (IRLC), in contrast, shows leaf complexity with over expression of KNOX1 gene, a finding attributed to FLO/LFY gene taking over this role (Champagne et al., 2007). Loss-of-function mutant of PALM1, by down-regulating expression of LEAFY/UNIFOLIATA orthologue SINGLE LEAFLET1 (SGL1), leads to five leaflets in *Medicago truncatula* instead of regular trifoliolate leaves (Chen et al., 2010). PALM1 is key regulator for dissected leaf morphogenesis among IRLC legume including *M. truncatula* (Chen et al., 2010) whereas its homologues together with KNOXI proteins regulate compound leaf development among non-IRLC legumes such as soybean and *Lotus japonicas* (Champagne et al., 2007). These PALM1 homologues were considered to have coevolved with the FLO/LFY orthologues in the IRLC legumes (Chen et al., 2010), which regulate the FLO/LFY-type transcription during compound leaf development (Champagne et al., 2007). Further studies revealed that formation of trifoliolate and diverse leaf forms in *M. truncatula* is controlled by the BEL1-like homeodomain protein PINNATE-LIKE PENTAFOLIATA1 (PINNA1) which represses transcription of the *LEAFY* (*LFY*) orthologue *SINGLE LEAFLET1* (*SGL1*) (He et al., 2020). Hence, PINNA1 orthologues have been speculated to be involved in compound leaf development (He et al., 2020). It is also reported that NAM/CUC3 genes are required for leaf dissection and leaflet formation in compound leaves among many species with inter-leaflet boundary (Blein et al., 2008). Hence, the role of different genes among the trifoliolate and stabilized pentafoliolate plant of *T. alexandrinum* can be further studied.

### Inheritance of multifoliolate trait in other crops

Occurrence of multifoliolate leaves and mode of inheritance have been reported among many crops. For example, the multifoliolate trait in alfalfa was considered to be readily transmitted and highly heritable (Bingham & Murphy, 1965; Hanson, 1972; Ferguson & Murphy, 1973; Brick, Dobrenz & Schonhorst, 1976) and two or more loci were considered to be involved in genetic control at the tetraploid level (Bingham, 1964). The trait in Lucerne has also been observed as somaclonal variation (Johnson et al., 1984). A distinct increase in the percentage of multifoliolate leaves occurred in crosses between multifoliolate plants as maternal parent and the trifoliolate cultivars. The multifoliolate character was reported to be heritable and the percentage of multifoliolate (mf) leaves can be increased through breeding and selection (Ford & Claydon, 1996). The Lucerne forms with high expression of the mf-mutation show increased biomass and biochemicals like saponins and antocyanins (Cherniavskih et al., 2019).

Multifoliolate leaf formation have been reported to be controlled by recessive allele or duplicate gene interaction in ground nut (Mouli & Kale, 1981; Vasanthi, 2003); dominance of lobed leaf shape over the entire or single recessive gene or single dominant gene in *Phaseolus aureus* (Chhabra, 1990; Veeraswamy & Kunjamma, 1958); monogenic recessive gene for trifoliolate leaf or two loci of genes in *Vigna radiate* (Bhadra, 1991; Soehendi et al., 2007); single recessive gene in *Vigna mungo* (Satyanarayana et al., 1989) and *Lf* gene controlling the five leaflet traits or the two loci gene in soyabean (Wang Heng et al., 2000; Soehendi et al., 2007).

Bingham (1964) postulated that genes at three independent loci controlled the production of multifoliolate leaves in the diploid material in Lucerne. A recessive gene (*mf*) in the homozygous condition at one locus was necessary for the production of multifoliolate leaves, while additive effects at two other loci were influenced by penetrance. These findings suggest that the multifoliolate trait is more prominent than the trifoliolate trait and the former can be increased and stabilized in the advancing generation through recurrent selfing.

In the above cited examples of multifoliolate leaf formation in various crops, different mechanisms of genetic control have been proposed. Here we present the hypothesis for pentafoliolate leaf formation in *T. alexandrinum* wherein we successfully developed true breeding leaf format (pentafoliolate), other than normal trifoliolate, among some of the plant progenies.

### Inheritance of multifoliolate trait in Trifolium

It is believed that the genus *Trifolium* originated from multifoliolate ancestors and that the number of leaflets was reduced during evolutionary time (Eames, 1961; Jaranowski & Broda, 1978; Zohary & Heller, 1984). The hypothesis gains strength by the fact that the evolutionary primitive species of *Trifolium* often have pentafoliolate leaves (Zohary & Heller, 1984; Ellison et al., 2006). The presence of a dominant locus that inhibits the expression of multifoliolate leaves, leading to trifoliolate leaves in white clover and the genetic control of trait by at least one gene on LG H1 was reported by Tashiro et al. (2010). This hypothesis supported the earlier premise that leaflet number suppressors result in lower leaflet numbers (Eames, 1961; Zohary & Heller, 1984).

Multifoliolate leaf alterations in a mutant population of red clover (*T. pretense*) were considered to be governed by at least three additive recessive pairs of alleles, together with some modifying genes of incomplete penetration (Jaranowski & Broda, 1978). The phenomenon of multifoliolate leaves in *T. pratense* and *T. incarnatum* was interpreted as an effect of reversible mutation (Malinowski, 1974) with little possibility to be genetically stabilized. The multifoliolate trait in red clover was reported to be preconditioned by homozygous recessive alleles at one or two loci (Simon, 1962) or determined by quantitative recessive trait (Taylor, 1982). Similarly, the multifoliolate trait in *T. incarnatum* L. was supposed to be controlled by a single recessive gene (Knight, 1969). In *T. repens* also the multifoliolate trait was mostly recessive without any Mendelian segregation pattern (Ford & Claydon, 1996) and environmentally influenced (Baltensperger, Wofford & Anderson, 1991; Tashiro et al., 2010). In contrast to such reports of recessive gene control of the trait, pinnate leaves of *Papilionaceae* are considered as an ancestral character and governed by dominant genes (Eames, 1961; Denffer et al., 1962). This reversal from dominant to recessive gene control in red clover was explained as a specific atavism to primitive forms through a series of alleles in the direction from dominants to recessives (Jaranowski & Broda, 1978).

Inheritance of leaflet number in Egyptian clover was believed to be controlled by five alleles and the leaflet number varying from three to seven, 1^3^ being a recessive allele and 1^7^ a dominant allele of the same gene (Ayub Agricultural Research Institute., 1967). Multifoliolate nature was stabilized as a pentafoliolate trait after several cycles of selfing and recurrent selection (Malaviya, Roy & Kaushal, 2009; Malaviya et al., 2012) and same was found in the present study where a few pentafoliolate plants were found to be true breeding in the year two. Multifoliolate character was stabilized in Lucerne also by means of several cycles of recurrent selection (Yankov, Yancheva & Petkova, 1995), although the population so developed contained a mixture of palmate (3–5 leaflets) and imparipinnate leaves (7–9 or sometimes 11 leaflets). Some of the trifoliolate plant progenies in our study showed expression of the multifoliolate trait, which might be a result of unmasking of trait. Bingham & Murphy (1965) also reported that some intercrossed multifoliolate progeny of alfalfa lacked penetrance for the multifoliolate condition in the first year. However, in the S1 population, plants with multifoliolate leaves emerged, although a higher percentage of multifoliolate plants was noted in the S_1_ population of multifoliolate individuals, suggesting higher gene dosage present in that parent for the multifoliolate condition.

Polyploidy has also been reported to increase the expression of multifoliolate leaf formation in Egyptian clover with increased vegetative growth (Khushk & Abidi, 1970). Pathak et al. (2015) noted that tetraploid plants developed from plants with near complete pentafoliolate diploid *T. alexandrinum* plants showed better expression of the pentafoliolate trait. Bingham (1964) postulated that two or more loci were involved in genetic control of multifoliolate leaf formation at the tetraploid level in Lucerne.

### Hypothesis for inheritance of multifoliolate trait in *T. alexandrinum*

The inheritance among a large number of plant progenies together with in vitro micro-propagation results did not establish a definite pattern of multifoliolate leaf formation, although the study established the presence of more than one leaf format in individual plants and a few progenies showing true breeding pentafoliolate trait. Expression of multifoliolate leaf formation was higher among the plants raised from seeds of multifoliolate plants. Multifoliolate and pure pentafoliolate plants occurred in the progenies of pure trifoliolate plants and vice-versa. There was an apparent increase in pentafoliolate leaf formation over the years after selection. In vitro axillary bud multiplication also confirmed that frequency of plantlets with multifoliolate leaf formation was higher when the bud was excised from the node with multifoliolate leaf. Hence, the discussion below focuses on the possibility of existence of heteroblasty, transposon and epigenetic control, in isolation or in combination, of multifoliolate leaf formation in *T. alexandrinum.*

The genetics of the multifoliolate trait in different crops, including *Trifolium,* has been presented in different ways as described above. The present results show some congruity with earlier findings but differ in that there are no previous reports of developing plant type with a different leaflet number other than the normal in any crop. Moreover, some of our results were far from indicative of a Mendalian inheritance; for example, the leaves with varying leaflet numbers developing on the same branch, i.e., chimeric sectors, reversal of leaf formats in both directions, and the 100% penetrance and expression of the pentafoliolate trait in a few plants progenies. Further, the number of leaflets is a discrete variable and cannot thus be considered a result of additive gene effect, producing a continuous variation.

The present study showed that the number of pentafoliolate leaves increases with node number and the same was true for hexafoliolate. The trend appeared to be a case due to heteroblasty, a phenomenon quite common among land plants. Heteroblasty is referred to a sudden morphological transition during development which may include internode length, stem structure, leaf form, size and arrangement (Zotz, Wilhelm & Becker, 2011). Sometimes heteroblasty is one possible “strategy” used by plants to cope with heterogeneous environmental conditions similar to phenotypic plasticity (Lloyd, 1984). The roles of Abiotic stressors in modifying ontogenetic trajectories have been well defined in *Acacia koa* with predicted pathway how the light and water availability has a role to play in heteroblasty by Rose, Mickelbart & Jacobs (2019). However, in the present study, the changes in leaflet number from trifoliolate to pentafoliolate and vice versa were occurring in the same plant or even in the different nodes of the same branch. The chimeric plants could be an example of heteroblasty, but the plants and the plant progenies observed with complete pentafoliolate leaves do not favour the heteroblasty to be an explanation of the phenomenon.

Present findings indicate the possibility of transposon or epigenetic control of the trait either independently or in interaction. Epigenetic changes also lead to phenotypic heritable variations without any alterations in the genome sequence, thus acts as a bridge between the phenotype and genotype of a cell. Epigenetic patterns can be stably inherited through mitosis and meiosis and could thus play a significant role in evolutionary processes (Baubec et al., 2010; Eichten et al., 2013). Morphological variation and phenotypic plasticity in *Arabidopsis thaliana*, especially under stress conditions, are reported to have significant epigenetic contribution, although such epigenetic variations are more likely to be reversible (Kooke et al., 2015).

Another possibility is that of role of transposon. Transposable elements (TEs), once considered junk DNA and genetic parasites, dominate the genomic landscapes of most plants and animals. TEs are now known to play important roles in genetic and epigenetic processes, leading to genome variation and influencing gene expression (Joly-Lopez & Bureau, 2014). Hence, the interpretation of the results is presented along the lines of a plausible TE explanation. The hypothesis proposes presence of class II DNA TEs that alter the expression of genes leading to variation in leaflet formation pattern. It was observed that there were frequent changes from trifoliolate to multifoliolate and their reversions. Thus, chimeric sectors were seen on individual branches of the plants. Out of total 14,767 events (Table 3) of leaf formation, a total of 2,440 (16.52%) were events of change. Of these events of change, 687 (28.16%) were reversal events. Such frequent change in leaf format can be attributed to TEs which brought the change in phenotype with change in its position on chromosomes. Although transposon studies have not been reported in *T. alexandrinum*, TEs are reported among other species of the genus. For example, a large number of TE families have been identified in white clover (Becker et al., 2016; Tayale’ & Parisod, 2013; Hand et al., 2010). Similarly, the genome of *T. pratense* has been reported to contain a larger repetitive portion and more abundant retrotransposons and DNA transposons (Ištvánek et al., 2014; Sato et al., 2005; De Vega et al., 2015). On the basis of the closer relationships between *Medicago truncatula* and red clover, it was presumed that the two species possess very similar TEs (Ištvánek et al., 2014). The change or reversal events, i.e., from trifoliolate to multifoliolate and vice versa, were effecting many a times change of single leaflet number, indicating that some sort of sequential slicing of genes was taking place. It is also possible that the transposons causing alteration in leaflet number are located near the centromere and get sliced to another part of the chromosome while undergoing mitotic division—a possible reason leading to true breeding pentafoliolates. Constitutive heterochromatic regions of the plant genome, such as peri-centromeric regions, knobs, and sub-telomeres, are reported to represent chromosomal niches heavily occupied by LTR-retrotransposons (Miller et al., 1998; Lippman et al., 2004; Kejnovsky et al., 2006). Highly repetitive regions like peri-centromeric and heterochromatic regions are known to be rich in TEs (Bennetzen & Wang, 2014). Centromeric satellites are intermingled with a particular group of gypsy-like elements called centromeric retrotransposons (CRs) (Kejnovsky, Hawkins & Ce’dric, 2012). However, when such slicing takes place during meiotic division, only after getting a chance to fertilize with a similar event in the other gamete, it can lead to homozygous condition and thus behave as true breeding. It has been repeatedly shown that certain insertions are heritable despite the possibility of such germ cells with TE insertion not producing a viable embryo (Liu et al., 2004). In the present study, multifoliolate plants were seen in the progenies of both trifoliolate and multifoliolate plants, although their frequency was much higher in the latter. Some plant progenies were also noted to have complete pentafoliolate nature and true breeding. This leads to the conclusion that the trait was homozygous among some of the plants through this process of TE moving through chromosomal rearrangement.

The third possibility could be the transposon acting together with epigenetic role in the control of transposable element transcription, replication and recombination. This appeared to be a plausible explanation to the progenies with near 100% expressivity of pentafoliolate leaves turning to progenies with 100% expressivity after second cutting. The temperature and sunshine differences between the growth conditions before cutting and that after second cutting appeared to more epigenetic changes to occur. DNA methylation, together with other chromatin modifications, is most often associated with silencing of transposable elements (TEs) (Kooke et al., 2015). Transposable elements exhibit significant intraspecific genetic and epigenetic variation in *Arabidopsis* (Underwood, Henderson & Robert Martienssen, 2017). Owing to TEs heterogeneity the basis of epigenetic control is a complex leading to the differential sensitivity of TEs to different epigenetic perturbations. Further, such modifications together with selection pressure define how the transposable elements can contribute genome evolution (Underwood, Henderson & Robert Martienssen, 2017). Some changes to the host plant due to TE proliferation is widely achieved by epigenetic silencing (Weil & Martienssen, 2008; Hu et al., 2010). Further, self-pollinators are supposed to be more efficient in suppressing TEs owing to more rapid fixation of epigenetic silencing patterns (Civan, Svec & Hauptvoge, 2011), although Bestor (1999) assumes that the aggressiveness of transposons in self-fertilizing sexuals is self-limited comparing to outcrossing sexuals, where the transposon fixation is nearly certain provided that the coefficient of selection imposed by the transposon is less than 0.5 when there is one or more transposition events per generation.

From the results, it is quite apparent that occurrence of odd number leaflets is always higher, and besides normal trifoliolate leaves, pentafoliolate leaves showed a tendency for stabilization. This event appears to be in the direction of evolution from a simple palmate leaf to a compound leaf, which is substantiated with the occasional occurrence of pinnate pentafoliolate leaves instead of palmate pentafoliolate leaves. Independent evolution of compound leaves from simple leaves said to be species-specific mechanisms (Efroni, Eshed & Lifschitz, 2010) and this may be the mechanism in this species. Elongated petiolules were also sometimes observed in white clover by Tashiro et al. (2010), which resulted in a frequently pinnate compound rather than a palmate compound. These multifoliolate pinnate leaves bear an even greater resemblance to the typical leaf morphology of legumes (Eames, 1961). These facts support the possibility of transposons fixing as a step towards evolution from simple to compound. This may be an example of invasion of TEs with evolutive significance as suggested by Rouzic & Capy (2005); however, in the present case, artificially imposed selection is also responsible for the genetic drift. It is possible that the putative TEs moved to a new place due to slicing off of TEs or part of the chromosome along with TEs during mitosis resulting in its fixing. The putative TEs might have down-regulated PALM transcription factor, resulting in altered leaf morphology such as pentafoliolate leaves. The phenomenon of TEs’ movement and conferring a novel function with a selective advantage for the host has been reported quite frequently (Feschotte & Pritham, 2007; Sinzelle, Izsvak & Ivics, 2009; Hoen & Bureau, 2012; Joly-Lopez & Bureau, 2014). The putative TEs which resulted in true breeding pentafoliolates may further evolve simply by replicating under phenotypic selection, as also postulated by Joly-Lopez et al. (2017). The proposed TE appears to be involved in the leaflet developmental process. Among plants, most ETEs are likely developmentally important transcription factors (Bundock & Hooykaas, 2005; Lin et al., 2007; Ouyang et al., 2011). TEs after being integrated into the genome and the chromosomal rearrangements might have become transpositionally inactive. Chromosomal rearrangements get fixed in the population by positive selection (Hoffmann, Sgro & Weeks, 2004; Coghlan et al., 2005; Orr, 2006; Rieseberg & Willis, 2007; Charlesworth, 2009), as observed in the present case. In the present study, selections were made for pentafoliolate trait which haslead it to homozygous condition. Tsukahara et al. (2009) and Jurka, Bao & Kojima (2011) also believed that a few TEs get fixed even in a small population due to selection pressure. At this stage, it is difficult to assess the number of TEs involved in leaflet formation. As the change in the plant phenotype is quite distinct, there may be more than one TE. Bursts of TEs were considered to lead to enhancement of the speciation process (Belyayev, 2014).

In addition to simply knocking out gene expression altogether, TE insertions can eliminate positive or negative regulatory functions. A classic example of this is the insertion of *Mutator* elements into a conserved non-coding sequence (CNS) in the first intron of the *knotted1* gene in maize (Greene, Walko & Hake, 1994). These insertions lead to ectopic expression of this gene in leaves. Given that changes in expression of *knotted1* homolog in various species are associated with differences in the degree of leaf lobing, it will be interesting to see how often these changes are associated with TE insertions into this CNS (Bharathan et al., 2002). The diverse functional impact of TEs, and their intrinsic contribution to genomic plasticity suggest that these elements play a major role in molecular diversification, and ultimately, in species divergence (Kejnovsky, Hawkins & Ce’dric, 2012).

Thus, the transposon hypothesis presented here appears the most plausible explanation for developing homozygous pentafoliolate trait in *T. alexandrinum*, however, further molecular studies may reveal more about the number and mode of transposon activity. To study the possible role of epigenetic regulation of transposons epigenetic recombinant inbred lines may also be required because the natural epigenetic variation is complicated due to the large contribution of DNA sequence variation within species as suggested by Johannes et al. (2009) and Reinders et al. (2009) to circumvent sequence variation. Among such lines there is a substantial reduction in DNA methylation, an increase in TE transcription rarely transposition of TEs (Tsukahara et al., 2009). Recent advances in genome-wide bisulphite sequencing also open up new opportunities (Schmitz et al., 2013; Eichten et al., 2013). Thus, the study provides new insight into a phenomenon occurring among many species that could lead to novel strategies for optimizing crop productivity through increasing photosynthetic area.

## Conclusion

Number of leaflet being a discrete variable, occurrence of multifoliolate leaves on individual branches (i.e., chimeric sectors), reversal events (i.e., from multifoliolate to trifoliolate), and then developing true breeding pentafoliolates were the factors leading to a hypothesis beyond normal Mendelian inheritance. Transposable elements (TEs) involved in leaf development were probably responsible for alterations in the expression of genes leading to the variations in number of leaflets formed. The putative TEs movement to a new place, owing to chromosomal rearrangements during mitosis, resulted in true breeding pentafoliolate trait. An odd number was observed to be preferred over an even leaflet number. The invasion of TEs with evolutionary significance was more effective due to selection and led to genetic drift. Occasional occurrence of pinnate pentafoliolate leaves appears to be directed by evolution from a simple to a compound leaf. The hypothesis provides new insight into understanding the genetic control of the trait considering the involvement of TEs in altering the expression of leaflet formation.

##  Supplemental Information

10.7717/peerj.10874/supp-1 Supplemental Information 1 Original data on the genetic phenotype

## Additional Information and Declarations

### Data Availability

The following information was supplied regarding data availability: Original data on the genetic phenotype are available as a Supplemental File.
